# The *Chironomus tentans* genome sequence and the organization of the Balbiani ring genes

**DOI:** 10.1186/1471-2164-15-819

**Published:** 2014-09-27

**Authors:** Alexey Kutsenko, Thomas Svensson, Björn Nystedt, Joakim Lundeberg, Petra Björk, Erik Sonnhammer, Stefania Giacomello, Neus Visa, Lars Wieslander

**Affiliations:** Department of Molecular Biosciences, The Wenner-Gren Institute, Stockholm University, SE 106 91 Stockholm, Sweden; Science for Life Laboratory, Department of Biochemistry and Biophysics, Stockholm University, SE 171 21 Solna, Sweden; Science for Life Laboratory, Department of Cell and Molecular Biology, Uppsala University, SE 752 37 Uppsala, Sweden; Department of Biochemistry and Biophysics, Stockholm University, SE 106 91 Stockholm, Sweden; Science for Life Laboratory, KTH, Royal Institute of Technology, Science for Life Laboratory, SE 171 65 Solna, Sweden

**Keywords:** Eukaryotic gene expression, Model organisms, Balbiani ring genes, Chromosome puffs

## Abstract

**Background:**

The polytene nuclei of the dipteran *Chironomus tentans* (*Ch. tentans*) with their Balbiani ring (BR) genes constitute an exceptional model system for studies of the expression of endogenous eukaryotic genes. Here, we report the first draft genome of *Ch. tentans* and characterize its gene expression machineries and genomic architecture of the BR genes.

**Results:**

The genome of *Ch. tentans* is approximately 200 Mb in size, and has a low GC content (31%) and a low repeat fraction (15%) compared to other Dipteran species. Phylogenetic inference revealed that *Ch. tentans* is a sister clade to mosquitoes, with a split 150–250 million years ago. To characterize the *Ch. tentans* gene expression machineries, we identified potential orthologus sequences to more than 600 *Drosophila melanogaster* (*D. melanogaster*) proteins involved in the expression of protein-coding genes. We report novel data on the organization of the BR gene loci, including a novel putative BR gene, and we present a model for the organization of chromatin bundles in the BR2 puff based on genic and intergenic *in situ* hybridizations.

**Conclusions:**

We show that the molecular machineries operating in gene expression are largely conserved between *Ch. tentans* and *D. melanogaster*, and we provide enhanced insight into the organization and expression of the BR genes. Our data strengthen the generality of the BR genes as a unique model system and provide essential background for in-depth studies of the biogenesis of messenger ribonucleoprotein complexes.

**Electronic supplementary material:**

The online version of this article (doi:10.1186/1471-2164-15-819) contains supplementary material, which is available to authorized users.

## Background

Species belonging to the family Chironomidae are widely distributed in areas with temperate climate, and they are important components in the food chains of freshwater lakes. The larvae live in the sediment, which is a repository for many persistent chemicals spread into water. Since several Chironomus species can be cultivated under laboratory conditions, they are extensively used for sediment toxicity tests, for example sensitivity to DDE, copper and silver [1–3]. In addition, the salivary glands of *Chironomus tentans* (*Ch. tentans*) constitute an exceptional experimental system for *in situ* analyses of gene expression.

Knowledge about gene expression in eukaryotes is central for the understanding of fundamental biological processes and disease mechanisms. Gene expression involves a series of sophisticated reactions that include the synthesis of a pre-mRNA and its packaging into a well organized pre-mRNA-protein complex (pre-mRNP), the accurate processing of the primary transcript into a mature mRNP, the export of the mRNP to the cytoplasm, and its translation into protein. These processes require the coordinated function of complex multi-component molecular machineries for example [4–9]. Biochemical and genetic studies in a large number of experimental systems have resulted in the identification and characterization of the individual components of such machineries, which include both proteins and RNAs. From these studies, we have learned that the molecules that make up the basic gene expression machineries are evolutionarily conserved for example [10].

Defining the individual components of the gene expression machineries has been the first step in the study of the gene expression pathway. The next essential steps involve studies of protein-protein and protein-RNA interactions aimed at defining the architecture of the machineries, as well as analyses of the function of the machineries and their components *in vivo*. Yet another important goal is to understand the regulatory networks that coordinate the different steps of gene expression. Successful efforts to study functions and mechanisms have been made, and in many instances these efforts have provided detailed structural and mechanistic information. Even so, many questions remain to be answered about the molecular processes of gene expression and about their regulation in the cellular context. A main obstacle is the shortage of methods to study the function of the gene expression machineries inside the cell. In this context, the Balbiani ring (BR) genes of *Ch. tentans* are extremely interesting because they constitute one of the few examples of active eukaryotic genes that can be visualized and analysed in the intact cell nucleus [11, 12].

The BR genes are expressed in the salivary gland cells of *Ch. tentans* larvae in a tissue-specific manner [13, 14]. Previous studies have proven the value of the BR genes for studies of different steps in gene expression, including studies of active chromatin and transcription [15, 16], pre-mRNA synthesis and pre-mRNP assembly [11, 17], processing of the pre-mRNA [18, 19], and nucleo-cytoplasmic transport of mRNPs [20–22]. The BR genes and their transcripts thus provide unique experimental opportunities for studies of the intranuclear events of gene expression.

An important prerequisite to perform analyses of gene expression in *Ch. tentans* is the access to sequence information for the individual components of the gene expression machineries. It is also important to relate the gene expression machineries in *Ch. tentans* to those present in other eukaryotes in order to assess the evolutionary conservation of the processes under study. With these specific goals in mind, we have determined the genome sequence of *Ch. tentans*. We have used transcriptome sequence data to aid in the identification of the *Ch. tentans* genes, and we have mined the *Ch. tentans* genome in search for genes that code for components of the gene expression machineries. We have also analysed the sequence and structure of the BR2 puff, and we provide a model for the organization of the chromatin bundles in the active BR2 locus. In summary, our results validate the generality of *Ch. tentans* as a model system for gene expression studies.

## Results and discussion

### Genome sequencing and sequence assembly

Genomic DNA extracted from a diploid epithelial cell line of embryonic origin [23] was used for the sequencing and assembly of the *Ch. tentans* genome. This cell line has been in culture for more than 20 years, and has been used extensively in research related to the study of transcription, pre-mRNA processing and mRNA-binding proteins [24–27]. The cell line genomic DNA potentially contains mutations introducing differences compared to organismal DNA. However, we focus our analyses on the identification of the C*h. tentans* genes. In this respect, the gene predictions are evidenced by RNAseq data using RNA extracted from different tissues and whole organisms at different developmental stages. Three different types of genomic DNA libraries were constructed and sequenced: Illumina paired-end (PE), Illumina 5 kb mate-pair (MP), and 454 single-end (SE). Sequence data amounting to a total of 12 Gb was obtained (Additional file 1: Table S1 and Table S2). A Kmer similarity analysis of quality-filtered reads indicated a relatively high level of heterozygosity, and an estimated size of about 200 Mb for the *Ch. tentans* genome (Additional file 1: Figure S1). Whole-genome shotgun assembly was performed with CLC (CLCbio, Aarhus, Denmark) using the Illumina PE reads and the 454 SE reads, which gave approximately 65,000 contigs with a total size of 180 Mb (Table 1). Post-assembly scaffolding with SSPACE [28] using the Illumina MP reads slightly increased the total assembly size to 213 Mb and improved dramatically the contiguity of the final assembly to an NG_50_ of 65 kb (Table 1). This estimate of the genome size is similar to previous measurements by microspectrometry [29], reassociation kinetics [30] and cytophotometry [31]. The total assembly size of 213 Mb is likely to be an overestimate due to the presence of allelic variants in the assembly, and thus we estimate that the size of the *Ch. tentans* genome is approximately 200 Mb.

**Table 1:** **Genome assembly statistics for** ***Ch. tentans***

	Contigs	Scaffolds
Number	64,342	26,025
Total size	180 Mb	213 Mb
Max size	163 Kb	756 Kb
NG_50_*	6.4 Kb	65 Kb
NG_80_*	1.2 Kb	15 Kb
Fraction of gapped sites	NA	16%
Mean gap size	NA	885 bp

*NG_50_ and NG_80_: length of the shortest size-ordered contig required to represent 50% and 80% of the estimated genome size, respectively.

The completeness of the assembled genome was evaluated by analyzing a set of 248 highly conserved core eukaryotic genes using hidden markov models (HMM) as implemented in CEGMA [32]. More than 97% of the core genes were scored as “complete” in the assembly (>70% aligned), and only one core gene was missing (<30% aligned), which indicates that the gene space is well represented in the assembly.

### Repetitive sequences in the *Ch. tentans*genome

Repeat analysis indicated that 10% of the assembled genome is repeated. This fraction was slightly higher, 15%, when assembly-independent quantifications were performed. These results are similar to reassociation kinetics data showing that 87% of the *Ch. tentans* genome sequence consists of single-copy DNA [30]. Minisatellites and low complexity repeats represent approximately one third of the repetitive fraction of the genome, while the remaining two thirds contain complex repeats. The complex repeats include DNA elements as well as very few LINEs (Long Interspersed Elements), SINEs (Short Interspersed Elements) and LTR (Long Terminal Repeat) elements. A considerable fraction of the complex repeats could not be reliably ascribed to any known family (Additional file 1: Table S3 and Table S4).

**Figure 1:**
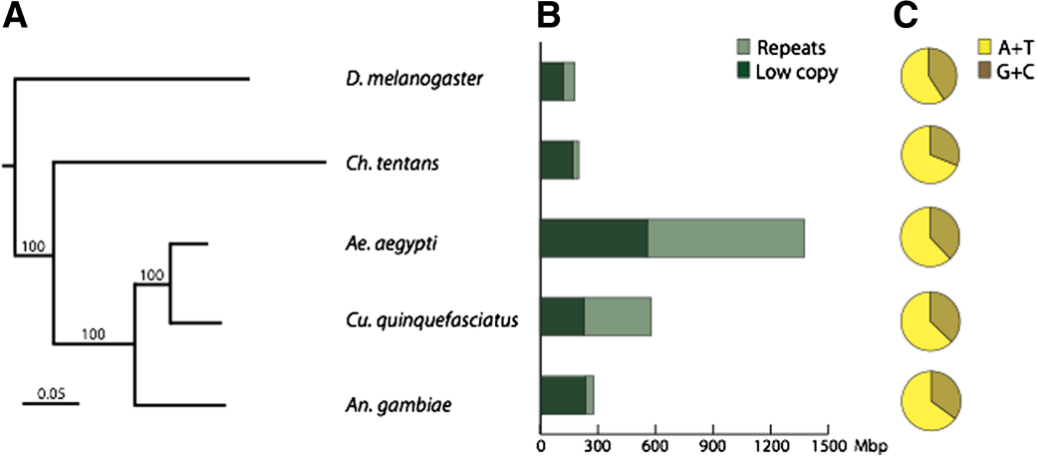
**Phylogenetic placement and genome characteristics of** ***Ch. tentans*** **compared to other Dipteran species. (A)** The phylogenetic placement as reconstructed from 531 conserved single-copy genes. The scale bar represents phylogenetic distance measured as nucleotide substitutions per site divided by the length of the sequence. **(B)** The genomic repeat content and **(C)** the genomic GC content of *Ch. tentans* and four other species of the order Diptera. The full phylogenetic reconstruction including nine arthropods and three outgroup nematodes is presented in Additional file 1: Figure S2.

The amount and nature of repetitive sequences varies considerably among the different Diptera [33], and the genome of *Ch. tentans* contains relatively few repetitive elements compared to some major exponents of its order (Figure 1B). Both the genome of *Aedes aegypti* (*Ae. aegypti*), the principal vector of yellow fever, and *Culex quinquefasciatus* (*Cu. quinquefasciatus*), the main vector of the nematode *Wuchereria bancrofti* have a high repeat content (about 55% and about 60%, respectively) and are relatively rich in transposable element [34–37], whereas *Ch. tentans* has very few transposable elements (Additional file 1: Table S3 and Table S4). Also transposable elements are well represented in the genome of *Anopheles gambiae* (*An. gambiae*), the major vector of malaria. Its euchromatic component contains 16% transposable elements, while its heterochromatin contains 60% transposable elements [38]. The heterochromatic component is characterized by 17% retrotransposons and a general poor expansion of short simple repeats (about 2%) [39]. This is in contrast to both the *Ch. tentans* genome, in which the fraction of simple repeats represents almost one third of the total repetitive fraction, and the *D. melanogaster* genome, in which simple repeats occupy a large part of the heterochromatin portion (one third of the whole genome) [40].

The genomes of some other Chironomus species contain a short tandem repetitive DNA element that is characterized by a Cla I-restriction site and therefore called “Cla-element” [41]. The Cla-element is able to transpose under certain circumstances, has a monomer length ranging from 110 to 119 bp, is very A + T-rich (>80% A + T), and displays numerous palindromic sequences [42]. The genomic concentration of Cla-elements is very different in closely related species and subspecies of Chironomus [43] and their localization in the genome are different [44]. Blastn analysis conducted on the *Ch. tentans* genome assembly and on a set of 454-reads did not identify any Cla-element sequences. This result is in accordance with previous Southern blot analyses, suggesting that the presence of Cla-elements is restricted in some Chironomus species [44].

### Transcriptome sequencing and genome annotation

We sequenced poly (A)^+^ RNA to characterize the *Ch. tentans* transcriptome. The RNA was extracted from a panel of eight samples that represented a variety of tissues and developmental stages. The RNA preparations were pooled and sequenced, and we obtained a total of 11.6 Gb in approximately 60 million raw read-pairs. The sequences were *de novo* assembled using Trinity, which gave initially 107,717 transcripts in 66,004 clusters (Table 2). With a cut-off at 98% of sequence identity, 69% of all the assembled transcripts could be fully aligned (>90% of their length) to the genome assembly, and 91% aligned partially (>30% of their length) within single scaffolds. We defined a set of 9,613 high-confidence (HC) transcripts by selecting the longest transcript per cluster and applying a quality filter that selected transcripts longer than 225 bp that were supported by at least 30 sequence reads.

**Table 2:** **The genome content and annotation of** ***Ch. tentans***

Genome	
Genome size	~200 Mb
Karyotype	2n = 8
GC content	31.2%
High copy repeat content	
Complex repeats	11%
Minisatellites/Low-complexity repeats	4%
Coding regions (excluding introns/UTRs)	9%
**Transcripts**	
Assembled transcripts (sequences/clusters)	107,717/66,004
High-confidence transcripts	9,613
**Annotation**	
Predicted coding gene loci	15,120
Fraction of genes with introns	83%
Average exon/intron size	312 bp/1,103 bp
Largest intron size	97 Kb
Fraction of short (<80 bp) introns	41%

UTR: Untranslated region.

The assembled RNAseq data was used as evidence in *ab initio* predictions of gene models and alternative splice variants (see Methods), which resulted in a set of 15,120 predicted genes. The majority of the genes, 83%, contained introns (Table 2). The obtained RNA sequences, when matched to the predicted genes, formed 35,424 different transcripts. Analyses showed that on average 2.3 transcripts represented each intron-containing gene, and that these transcripts were splice variants. The average number of introns per gene was 2.8. The average exon length was 312 bp, which was shorter than the average intron length (1,103 bp), and the length of the longest intron exceeded 97 kb. The total gene space including predicted introns and untranslated regions (UTRs) comprised 70.6 Mb, which corresponds to 36% of the estimated genome size. The coding regions covered 17.9 Mb or 9% of the estimated genome size (Table 2).

A large fraction (41%) of the introns in the *Ch. tentans* genome were very short (<80 bp), a feature that was previously also noted in *D. melanogaster* [45, 46]. Different mechanisms for intron recognition and spliceosome assembly have been described that depend on the length of the introns and exons involved in the splicing reaction. The abundance of very short introns in *Ch. tentans* suggests that the so-called *intron definition* mechanism, a mechanism of intron recognition based on interactions between the 5’ and 3’ splice-sites across the intron [47], is a major mechanism for spliceosome assembly in *Ch. tentans*.

The average GC content for the *Ch. tentans* genome is 31%, which is somewhat lower than that of other Dipterans such as *An. gambiae* (35%) [38] or *D. melanogaster* (41%) [40] (Figure 1C). The GC content of the coding regions, 37%, was considerably higher than the average for the genome, whereas the introns and the intergenic regions were characterized by lower GC contents, down to 30%.

The *Ch. tentans* genes were classified into gene families using the OrthoMCL-DB database [48], an orthology database that comprises protein-coding genes from 150 species, including nine arthropods (Additional file 1: Table S5). The OrthoMCL-DB analysis could align 12,234 genes (81%) of *Ch. tentans* to 7,111 previously identified gene families, with the remaining 2,886 species-specific genes (19%) being either contained in new in-paralog gene families (7%) or classified as orphan genes (12%). These figures are similar to those obtained for the well-annotated model species *D. melanogaster* whose genome contains 8,349 (74%) cross-species gene families and 26% species-specific genes according to the OrthoMCL-DB. Out of the 7,111 *Ch. tentans* gene families, 5,620 families (79%) were shared with the three mosquito genomes included in the analysis (*An. gambiae*, *Ae. aegypti* and *Culex quinquefasciatus (Cu. quinquefasciatus*), and as many as 6,853 families (96%) were shared with at least one of these.

We performed a phylogenetic reconstruction based on a set of 531 conserved single-copy genes across the nine arthropods and three nematodes included in the orthoMCL-DB database and the present *Ch. tentans* sequence. We show that *Ch. tentans* represents a sister clade to mosquitoes (Figure 1 and Additional file 1: Figure S2). The split to mosquitoes dates back roughly 150–250 million years [49].

### The gene expression machineries of *Ch. tentans*

**Figure 2:**
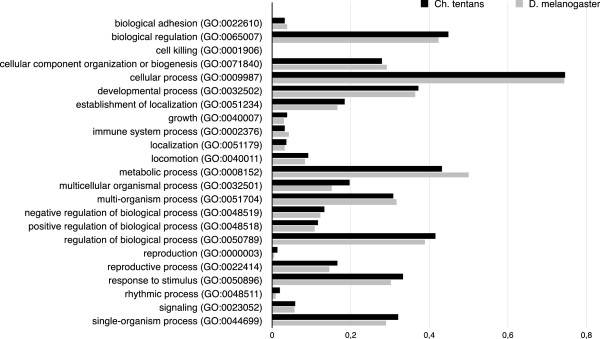
**Comparison of the relative abundance of proteins according to gene ontology terms.** Gene ontology categories of “biological process” GO-domain were used for annotation of the *Ch. tentans* proteome and compared to gene ontology annotation of the *D. melanogaster* proteome. The bars represent the relative abundance of GO categories, i.e. for every dataset we calculated the frequencies of annotations of proteins to a GO category relative to the total amount of proteins annotated to “biological processes”.

Gene onthology analyses were performed on the predicted protein-coding genes of *Ch. tentans* using Blast2Go [50] based on blasting the sequences against *D. melanogaster* proteins (The FlyBase Consortium: FlyBase. 1993–2014 (http://flybase.org)). An interspecies comparison of GO categories within “biological function” domain showed that the relative frequencies of each category in *Ch. tentans* were very similar to those found in *D. melanogaster*, as expected (Figure 2).

**Figure 3:**
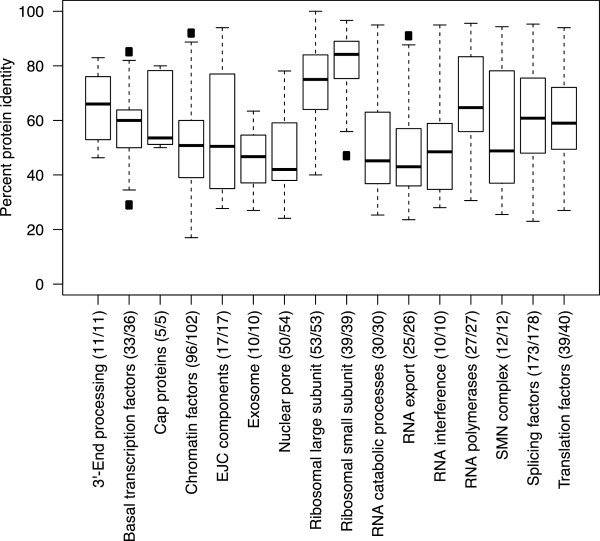
**Conservation of gene expression machineries in** ***Ch. tentans*** **.** Boxplot of the percentual protein identity between potential orthologous sequences in *Ch. tentans* and *D. melanogaster* for 16 gene expression machineries. The proteins in each machinery were identified in *D. melanogaster* and aligned to the *Ch. tentans* genome and transcriptome assemblies, excluding alignments covering less than 25% of the *D. melanogaster* protein length. The aligned and total number of proteins is shown for each machinery. Filled black rectangles represent outlier values in the distributions.

Given the relevance of *Ch. tentans* as a model system for studies of gene expression, it was important to identify the components of the gene expression machineries in *Ch. tentans* and to establish their degree of evolutionary conservation. To this end, we defined the main processes included in the gene expression pathway, listed the known components of the corresponding machineries in *D. melanogaster* (16 machineries comprising 664 proteins), and identified their orthologs in *Ch. tentans* as follows. The protein sets for the 16 expression machineries were compiled in *D. melanogaster* (FlyBase r5.55) by expert annotation (Additional file 2). A complete set of orthologs between *D. melanogaster* and *Ch. tentans* proteins were identified by InParanoid [51]. From these, 407 1:1 orthologs of the 16 machineries were extracted and aligned using Kalign v1.04 [52]. Each *D. melanogaster* protein was also aligned by Exonerate v 2.2.0 [53] and by tblastn (Blast 2.2.25), to both the complete genome assembly and the set of HC transcripts. In total, a maximum of 5 protein identity scores were thus obtained per protein, and the highest score was kept as an estimation of the *D. melanogaster* versus *Ch. tentans* protein conservation. After manual inspection of hits and selection of alignments covering at least 25% of the *D. melanogaster* proteins, potential orthologs were identified for 649 of the 664 proteins included in the study (Additional file 1: Table S6), with percentages of protein identity ranging from 100% (*RpL41*) to 17% (*Asx*) (Figure 3, Additional file 2). The large and small ribosomal subunits were the most highly expressed and the most highly conserved machineries, but otherwise no general correlation was observed between protein conservation and gene expression level in this dataset (Additional file 1: Figure S3 and Additional file 3). Most of the proteins in *D. melanogaster,* for which no orthologs could be identified in *Ch. tentans* (Additional file 1: Table S6), are proteins with relatively low degree of conservation that lack orthologs outside the genus *Drosophila* according to the OrthoDB catalogue [54]. Two of them, Ulp1 and Trf2, have orthologs in yeast and metazoans, and are therefore likely to exist also in *Ch. tentans*. These two proteins are unusually long, their sequence conservation is not high, and the homology is restricted to parts of the sequence, which could be the explanation why no orthologs were identified in our study.

We also searched the *Ch. tentans* genome with full-length *D. melanogaster* snRNA sequences to identify the spliceosomal U1, U2, U4, U5 and U6 snRNA genes in the *Ch. tentans* genome. In all cases, we found matches over at least 55% of the full length of the *D. melanogaster* sequences, with identities in the matched regions ranging between 77% and 100% (Table 3 and Additional file 1: Table S7). A comparison between the *Ch. tentans* and human snRNA sequences revealed a similar degree of identity (data not shown). We conclude that the *Ch. tentans* genome contains a complete set of the major spliceosomal snRNA genes.

**Table 3:** **The snRNA genes of** ***Ch. tentans***

snRNA gene	nr genes in ***Ch. tentans***	nr. genes in ***D. melanogaster***	% identity in matched regions
**U1**	2	5	80
**U2**	1	6	83-84
**U4**	2	3	77-80
**U5**	3	7	84-91
**U6**	3	3	97-100

In summary, our analysis shows that the gene expression machineries encoded in the *Ch. tentans* genome are highly conserved, which is of relevance for the studies of gene expression in this model organism.

### Sequence organization of the BR gene loci

Previous studies of gene expression using *Ch. tentans* focused on the BR genes [11, 12] and early sequence analyses provided a partial view of the BR gene family [14]. Four BR genes (BR1, BR2.1, BR2.2 and BR6) are approximately 40 kb long, have a similar exon-intron structure with four introns, and share internal repetitive sequence organization. The BR3 gene is 11 kb long and is related to the long BR genes, but contains 38 introns spread throughout the gene and has a diverged internal repetition [55].

**Figure 4:**
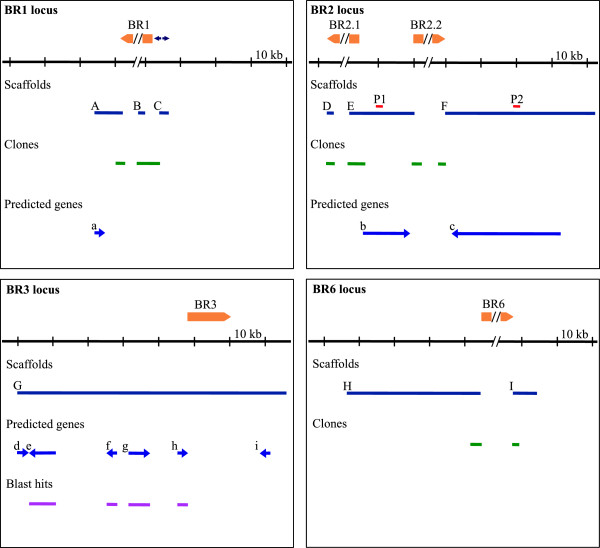
**The BR gene loci of** ***Ch. tentans*** **.** The sequence organization of the BR1, BR2, BR3 and BR6 gene loci are shown schematically. Solid blue lines show scaffolds (indicated by capital letters). Previously cloned gene fragments are shown in green. Predicted genes are shown by dark blue arrows and labelled by small letters (direction of the genes are indicated by the arrowheads). Blast hits are shown in pink. In the BR3 locus, the blast hits corresponding to the predicted genes were; gene e: protein FBpp0086723, gene f: protein FBpp0289635, gene g: protein FBpp0075699, gene h: protein FBpp0084614. The positions of the BR genes, in relation to the scaffolds, are shown in yellow in the upper parts of the the images. Each gene (except BR3) is interrupted in the middle. The interruption corresponds to the approximately 35 kb repetitive central part of each gene. Below the BR genes, the black line serves as a length marker. In the BR1 locus, the two purple arrows in opposite direction upstream the BR1 gene represent the palindromic upstream sequences. In the BR2 locus, P1 and P2 (in red) indicate the positions of the probes used for *in situ* hybridization.

Here, the current genome assembly combined with sequence information from previously cloned genomic fragments were used to describe the non-repetitive parts of the BR genes, providing novel information on the genome organization of the BR genes (Figure 4). The central coding regions of the BR1, BR2.1, BR2.2 and BR6 genes are built from tandem repeats and were not recovered in the genome assembly, except for a limited number of repeats located at both ends of these regions. The sequences of these four genes were therefore recovered in two or three scaffolds, while in the BR3 locus, the entire BR3 gene was present in a single scaffold. In the BR1 locus, a 2.8 kb-long promoter region was duplicated, in reverse orientation, with a sequence of about 300 bp between the palindromic sequences (Figure 4, BR1 locus). PCR analyses confirmed that the BR1 gene is connected to the promoter-containing scaffold as indicated in the figure, while the presence of a putative second BR gene, in the BR1 locus, connected to the duplicated promoter could not be determined at this stage. Previously, an additional internally repetitive gene fragment from the BR1 locus has been described [56], but we were unable to connect this gene to the duplicated promoter using PCR. A short gene, located approximately 7 kb downstream the BR1 gene was predicted, but no orthologs were found in Blast searches. No additional genes could be mapped in the BR1 gene locus.

In the BR2 locus, the closely related BR2.1 and BR2.2 genes turned out to be located approximately 15 kb apart and in opposite directions (Figure 4, BR2 locus). The sequences in the 200 bp immediately upstream the transcription start sites were essentially identical for the two genes. In between the two genes, several regions, 1–1.5 kb in length, had palindromic organization with 55-80% sequence identity. These regions were present close to the BR2.1 gene and in the middle of the intergenic region. The functional significance of these palindromes is not known. We predicted one gene located between the two BR2 genes and a second gene downstream the BR2.2 gene. The major parts of these two predicted genes consisted of proposed non-coding sequences and only short coding regions were present. The expression of these predicted genes remains to be experimentally demonstrated.

In the BR6 locus (Figure 4, BR6 locus), we found the beginning and end of the BR6 gene in two scaffolds. No additional genes were present in the scaffolds.

The BR3 gene, (Figure 4, BR3 locus), is located close to several other genes. In the upstream, approximately 47 kb region, five predicted genes are present. Four of these have orthologs in *D. melanogaster*. In the downstream region, a short predicted gene is present, but no Blast hit could be found.

In the genome assembly, a previously unknown BR-like gene was discovered (Additional file 1: Figure S4A). The promoter region and the beginning of the gene, including transcription and translation start sites and exon 1, intron 1 and the beginning of exon 2, were very similar to the corresponding regions in the BR1, BR2.1, BR2.2 and BR6 genes. Also the 3′ end of the gene was very similar to the long BR genes. At present, we do not know if the central part of this BR-like gene contains repeats similar to the previously characterized BR genes. *In situ* hybridization located this BR-like gene to locus 5B on chromosome IV (Additional file 1: Figure S4B). This locus is not unfolded into a morphologically visible BR puff in the salivary gland polytene chromosome IV, which suggests that the gene is not highly transcribed and/or that the gene locus is not organized as the BR1, BR2 or BR6 loci are.

### The organization of the BR2 puff

**Figure 5:**
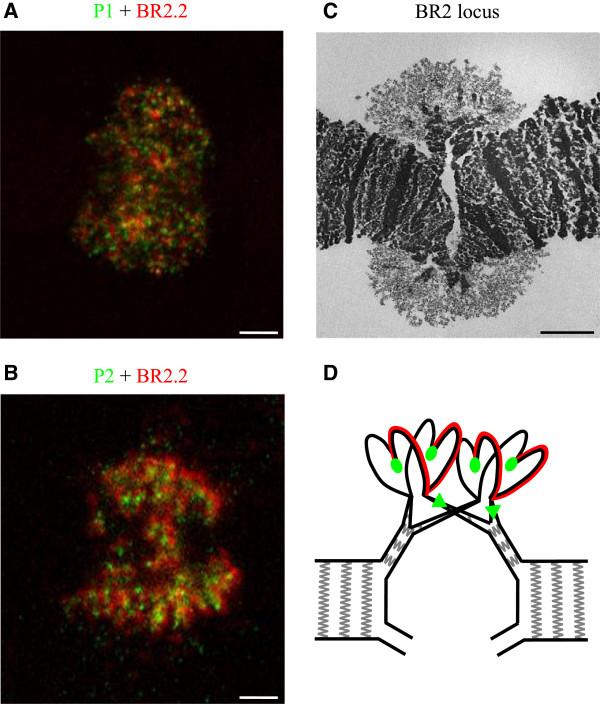
**The organization of the BR2 gene locus. (A-B)** A probe representing the repetitive coding sequence of the BR2.2 gene (red) was hybridized simultaneously with either the intergenic probe P1 **(A)** or the downstream probe P2 **(B)**, both in green. **(C)** A thin section through the BR2 gene locus visualized in a transmission electron microscope. **(D)** A model for the organization of the chromatin bundles in the BR2 puff. The polytene chromosome is split into many gradually thinner chromatin bundles. From thin bundles, individual chromatids are unfolded extensively along the transcribed BR2.1 and BR2.2 genes. The intergenic region (detected by *in situ* hybridization with the P1 probe, depicted as oval green signals) is refolded into more compact chromatin, but is still located in the periphery of the gene locus. From the intergenic region, the transcribed part of the BR2.2 gene is unfolded. The red line symbolizes the *in situ* hybridization signal for the coding region of the BR2.2 gene, visualized with the repetitive probe. At the 3′ end of the BR2.2 gene, the downstream chromatin is compacted and gradually interact with regions from other chromatids to form thicker and larger bundles. The downstream hybridization probe signal (green triangle) labels such chromatin bundles that are more centrally located in the puff. The bars represent 5 μm in A, B and C.

The transcribed BR genes are extensively unfolded and form giant puffs in the polytene chromosomes [11], but little is known about the topological organization of the BR genes and their flanking sequences in the puffs. We have made use of the genomic sequence data to visualize and analyze the organization of the two BR2 genes in the active BR2 locus. We performed *in situ* hybridizations using probes representing the region between the BR2.1 and BR2.2 genes (probe P1 in Figure 4, BR2 locus), the BR2.1 and BR2.2 repetitive coding regions and the downstream region of the BR2.2 gene (probe P2 in Figure 4, BR2 locus). The P1 intergenic probe labelled distinct spots at many sites throughout the entire BR2 locus (Figure 5A). These spots did not overlap with the probes for the transcribed repetitive regions of the BR2.1 (data not shown) or BR 2.2 genes (Figure 5A), both of which labelled more extended and peripheral regions. The P2 probe located downstream the BR2.2 gene also hybridized to spots located in a scattered manner throughout the BR2 puff (Figure 5B). However, these spots were fewer and larger than the ones seen for the P1 intergenic region, and they were more internally located.

Electron microscopy (EM) images of thin sections through the BR2 locus (Figure 5C), [15] reveal that the BR2 locus contains chromatin bundles of gradually thinner dimensions that extend from, and return to, the chromosome axis. It is also known that the active BR genes form transcription loops with an extended chromatin axis [57]. However, the BR2.1 and BR2.2 genes could not be distinguished from each other by EM morphological criteria, and the organization of the two genes inside the puff was unknown. Our *in situ* hybridization results show that the two transcribed genes are intermingled in the BR2 puff, as shown by the fact that the probes specific for the BR2.1 and BR2.2 repeats give very similar staining patterns over the entire puff.

The BR2.1 and BR2.2 repetitive probes gave a diffuse hybridization signal, whereas the P1 intergenic probe gave a spotted staining. This difference suggests that only the transcribed parts of the genes (which consist mostly of repetitive sequences) loop out from the chromatin bundles in an extended conformation. The intergenic sequences are instead packaged in more compact chromatin patches and are therefore visualized as spots by *in situ* hybridization.

Detailed EM studies showed that the chromatin located immediately upstream of the transcribed BR gene forms a thin chromatin fibre corresponding to about 0.5 kb of DNA [15]. Several such fibres representing several genes extend from a single compact chromatin region. Our *in situ* hybridization results are compatible with the EM ultrastructure and suggest that the promoter-containing intergenic region forms thin bundles of condensed chromatin engaging several chromatids, and that these bundles are located at the periphery of the chromosome body (Figure 5D).

In the EM, the downstream region appears as a loosely coiled chromatin fibre, 200 nm in length, corresponding to about 3 kb of DNA [15]. Several such chromatin fibres connect to a more compact chromatin patch. Our *in situ* hybridization data are consistent with the interpretation that sequences located about 20 kb downstream the BR2.2 gene reside in compact chromatin. The fact that the downstream probe P2 labels fewer spots than the upstream probe P1, suggests that the downstream sequences from many chromatids (more chromatids than for the upstream region) come together in more compact chromatin bundles (Figure 5D). Moreover, these downstream chromatin bundles are located more internally than the intergenic bundles visualized with the P1 probe. This difference suggests that the bundling of chromatids at the two ends of the BR genes is controlled by different molecular mechanisms.

## Conclusions

The *Ch. tentans* genome is approximately 200 Mb in size, contains 15% repetitive elements, and encodes 15,120 genes with an average of 2.3 alternative transcripts per gene. We have identified genes that code for factors involved in the expression of protein-coding genes, including snRNA genes, and we show that these factors are similar to their orthologs in *D. melanogaster*. We also report novel data on the organization of the BR gene loci, the identification a novel putative BR gene, and present a model for the organization of chromatin bundles in the BR2 puff. Our results strengthen the generality of the BR genes as a model system and provide essential background for in-depth studies of mRNP biogenesis and functions using a unique eukaryotic model system. The access to the *Ch. tentans* genome sequence will not only ease research in the field of gene expression but also constitute a valuable resource for toxicity and ecology studies. The analysis of the *Ch. tentans* genome sequence will also contribute to studies of genomic evolution of Nematocera and of other insects that are relevant for human activity and health, such as mosquitoes, blackflies and sandflies.

## Methods

### Genome sequencing and assembly

Genomic DNA was isolated from an embryonic epithelial *Ch. tentans* cell line [23]. Cells were pelleted by centrifugation and suspended in 5 ml 10 mM Tris–HCl (pH 8.0), 10 mM EDTA. 10 mM NaCl, 250 μl of 10% SDS and 2 mg of Proteinase-K were added followed by 37°C over-night incubation. The DNA was phenol extracted twice and the final water phase was dialyzed against 50 mM Tris–HCl (pH 8.0) containing 10 mM EDTA. 10 mM NaCl, RNase A (50 μg/ml) and RNase T1 (2 μg/ml) were added and the extract was incubated at 37°C for 30 minutes, followed by phenol extraction twice. 1/10 volume of 3 M Sodium Acetate (pH 5.2) and 2.5 volumes of ethanol were added and the DNA was recovered on a glass rod used to gently mix the water and ethanol phases. The DNA was finally dissolved in 10 mM Tris (pH 8.0), 1 mM EDTA. The cell line genomic DNA had a size greater than 50 kb and a high degree of purity.

Whole genome shotgun sequence data was obtained from a 500 bp insert library (Illumina, 2 × 100 bp, 23× coverage), a 5 kb mate-pair (“jumping”) library (Illumina, 2 × 44 bp, 22× coverage), and a 454 library (45, 1 × 400 bp, 4× coverage) (Additional file 1: Table S1). Illumina sequences were obtained from BGI (Shenzhen) and 454 sequences from SciLifeLab (Stockholm). The reads were quality-filtered, adaptor sequences were trimmed, and duplicate reads were removed before use. Contamination analyses were performed using a random 5% subset of the reads blasted against the NCBI nucleotide database (Additional file 1: Table S2). The genome was assembled with CLCbio v5.5.1, kmer = 24 nt, (CLCbio, Aarhus, Denmark), followed by scaffolding with SSPACE v 1.0 [28]. For scaffold pairs likely representing heterozygous alleles (≥98% identity across ≥ 95% of the length), the smaller scaffold was removed from the assembly. An assembly-independent genome size estimation was performed based on kmer analysis of quality filtered Illumina PE reads in Jellyfish [58] (Additional file 1: Figure S1).

### Analysis of repetitive sequences

An *ab initio* species-specific repeat library was constructed and classified with RepeatModeler v1.0.7 [59], and further classified using 14 repeat sequences belonging to *Ch. tentans* and its sibling species *Ch. pallidivittatus* (Additional file 1: Table S3). Minisatellite (10–99 bp, repeated at least 2.9 times) and satellite (>100 bp, repeated at least 2.9 times) sequences were detected using TRF [60] and clustered by cd-hit [61]. To quantify the genomics repeat content, the resulting repeat library (Ctentans_rep_library_v1.0) was used as input for RepeatMasker [62] to mask both the assembly and a subset of 180,000 454 reads of length >500 bp (Additional file 1: Table S4).

### Transcriptome sequencing, annotation and gene family analyses

Total RNA was extracted from the same cell line used for DNA genome sequencing [23], and from different tissues and developmental stages of *Ch. tentans*: egg strings, complete first instar larvae, salivary glands, gut, nervous system and fat bodies from fourth instar larvae, and complete imagos. The samples were homogenized in Trizol and total RNA was extracted with a Trizol-chloroform mixture as indicated by the manufacturer (Invitrogen). The RNA was precipitated with 100% isopropanol and the pellet was washed with 75% ethanol. The washed RNA pellet was air-dried and finally dissolved in water. The integrity of the RNA was checked by agarose gel electrophoresis.

Equal amounts (4 μg) of RNA from each source were pooled, and poly (A)^+^ RNA was isolated, reverse transcribed into cDNA and paired-end sequenced (Illumina, 2 × 101 bp) (SciLifeLab, Stockholm). 10.5 Gb from 56.8 million quality-filtered read pairs were *de novo* assembled using Trinity [63]. A subset of 9,613 high-confidence transcripts (from the total set of 107,717) was selected by removing all transcripts shorter than 225 bp or with an average read coverage below 30×.

Gene models were predicted with Augustus v 2.6.1 [64]. The predictions were run three times using different input evidence datasets: 1) the 9,613 high-confidence Trinity assembled transcripts (see above), 2) all Trinity assembled transcripts, and 3) no assembled transcripts. The prediction using the high-confidence transcript assemblies resulted in 10,096 predicted gene loci. From predictions 2) and 3), we selected only non-overlapping gene loci, which, in addition showed similarity to the protein database (NCBI, NR). Eventually, gene loci from the predictions using all assembled transcripts (adding 4,818 gene loci) and from the predictions using no assembled transcripts (adding 206 gene loci) were added to the initial set. The final set of predicted genes thus contained 15,120 gene loci.

All protein-coding genes were classified into protein families based on orthoMCL-DB v5 [46] using the longest predicted protein sequence for each gene loci. A phylogenietic tree (Additional file 1: Figure S2) was reconstructed (RAxML v7.2.8, PROTGAMMAJTTF model, 100 bootstrap replicates [65]) based on a concatenated alignment (Kalign v2.04) [52] of 531 single-copy core genes (Additional file 4), conserved across all the ten arthropods and the three outgroup nematodes included in the analysis (Additional file 1: Table S4). Uncertain positions were screened away with Gblocks v 0.91b [66].

### BR gene loci analyses

The assembled *Ch. tentans* genome scaffolds were searched with previously determined BR gene sequences [14], with critical regions of the gene loci verified by PCR, or RT-PCR. Upstream and downstream regions of BR genes were amplified from genomic DNA by PCR (oligonucleotide primers used for PCR, see Additional file 1: Table S8) and served as probes for *in situ* hybridization, using squash preparations of salivary gland polytene chromosomes [67]. The sequences of the BR2.1 and BR2.2 coding repeat units were detected using labelled oligonucleotides as hybridization probes. Preparations were visualized and photographed using either a Zeiss Axioplan II fluorescence microscope or a Zeiss 510 META confocal microscope.

### Transmission electron microscopy

Polytene chromosomes were isolated and processed for EM as previously described [68]. EM preparations were viewed and acquired at 80 kV in a 120 kV Tecnai electron microscope (FEI), using a charged-coupled device camera (1000P, Gatan) and The Digital Micrograph acquisition software (Gatan).

### Data access

The draft genome assembly of *Ch. tentans* are provided under the accession numbers HG428765-HG454789 (EBI project number PRJEB1888).

## Electronic supplementary material

Additional file 1: **Contains the following items:** **Figure S1.** Kmer coverage frequency histogram of quality filtered PE Illumina reads. **Figure S2.** A maximum-likelihood phylogenetic tree reconstructed from a concatenation of 531 core gene protein alignments. **Figure S3.** Gene expression values in *Ch. tentans* (log FPKM) versus percent protein identity between potential *D. melanogaster* and *Ch. tentans* orthologs for 16 expression machineries. **Figure S4.** A predicted novel BR gene. **Table S1.** Statistics on genome sequencing libraries. **Table S2.** Species distribution of sequences in the NCBI nucleotide database (nt) with homology against a 5% random subset of *Ch. tentans* sequencing reads. **Table S3.** Previously identified *Chironomus* repeat sequences added to the *Ch. tentans ab initio* repeat library. **Table S4.** The repeat content of the *Ch. tentans* genome. **Table S5.** Species included in the OrthoMCL-DB gene family analysis and the phylogenetic reconstruction. **Table S6.** Expression machinery genes in *D. melanogaster* with no detected orthologous sequence in *Ch. tentans*. **Table S7.** The U snRNAs of *Ch. tentans*. **Table S8.** Oligonucleotides used for PCR and *in situ* hybridization experiments. (DOC 787 KB)

Additional file 2: **Expression machineries.** (XLS 270 KB)

Additional file 3: **Omniscope data file corresponding to Additional file** 1. Figure S3, which can be viewed interactively with Omniscope Viewer (http://visokio.com/download). (ZIP 128 KB)

Additional file 4: **Multiple sequence alignments for the 531 single-copy core genes, before screening of uncertain positions.** (ZIP 2 MB)

## Abbreviations

Bp: Base pair
BR: Balbiani ring
PCR: Polymerase chain reaction
RNP: Ribonucleoprotein
EM: Electron microscopy.
