# Supplementary material for: The Chironomus tentans genome sequence and the organization of the Balbiani ring genes
Source: BMC Genomics. 2014 Sep 27;15(1):819. doi: 10.1186/1471-2164-15-819 (PMC4192438; doi:10.1186/1471-2164-15-819)
Supplement: Supplementary file 1 — Additional file 1: Contains the following items: Figure S1. Kmer coverage frequency histogram of quality filtered PE Illumina reads. Figure S2. A maximum-likelihood phylogenetic tree reconstructed from a concatenation of 531 core gene protein alignments. Figure S3. Gene expression values in Ch. tentans (log FPKM) versus percent protein identity between potential D. melanogaster and Ch. tentans orthologs for 16 expression machineries. Figure S4. A predicted novel BR gene. Table S1. Statistics on genome sequencing libraries. Table S2. Species distribution of sequences in the NCBI nucleotide database (nt) with homology against a 5% random subset of Ch. tentans sequencing reads. Table S3. Previously identified Chironomus repeat sequences added to the Ch. tentans ab initio repeat library. Table S4. The repeat content of the Ch. tentans genome. Table S5. Species included in the OrthoMCL-DB gene family analysis and the phylogenetic reconstruction. Table S6. Expression machinery genes in D. melanogaster with no detected orthologous sequence in Ch. tentans. Table S7. The U snRNAs of Ch. tentans. Table S8. Oligonucleotides used for PCR and in situ hybridization experiments. (DOC 787 KB) [file 12864_2014_6510_MOESM1_ESM.doc]

**The *Chironomus tentans* genome sequence and the organization of the Balbiani ring genes**

**ADDITIONAL FILE 1:**

**SUPPLEMENTARY FIGURES**

**
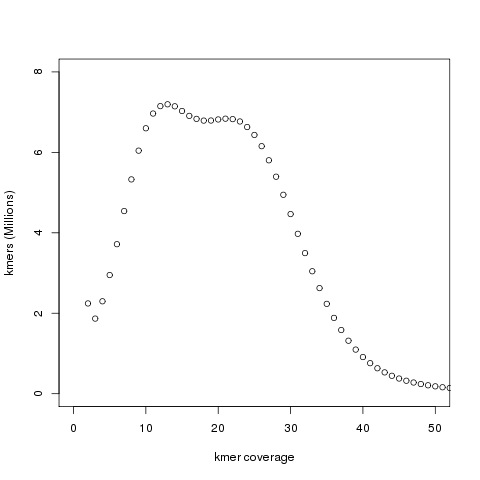
**

**Figure S1.** Kmer coverage frequency histogram of quality filtered PE Illumina reads. The total number of kmers (k=27 bp) was 4145961139, and the main peak was estimated from the histogram to 21X kmer coverage.

**
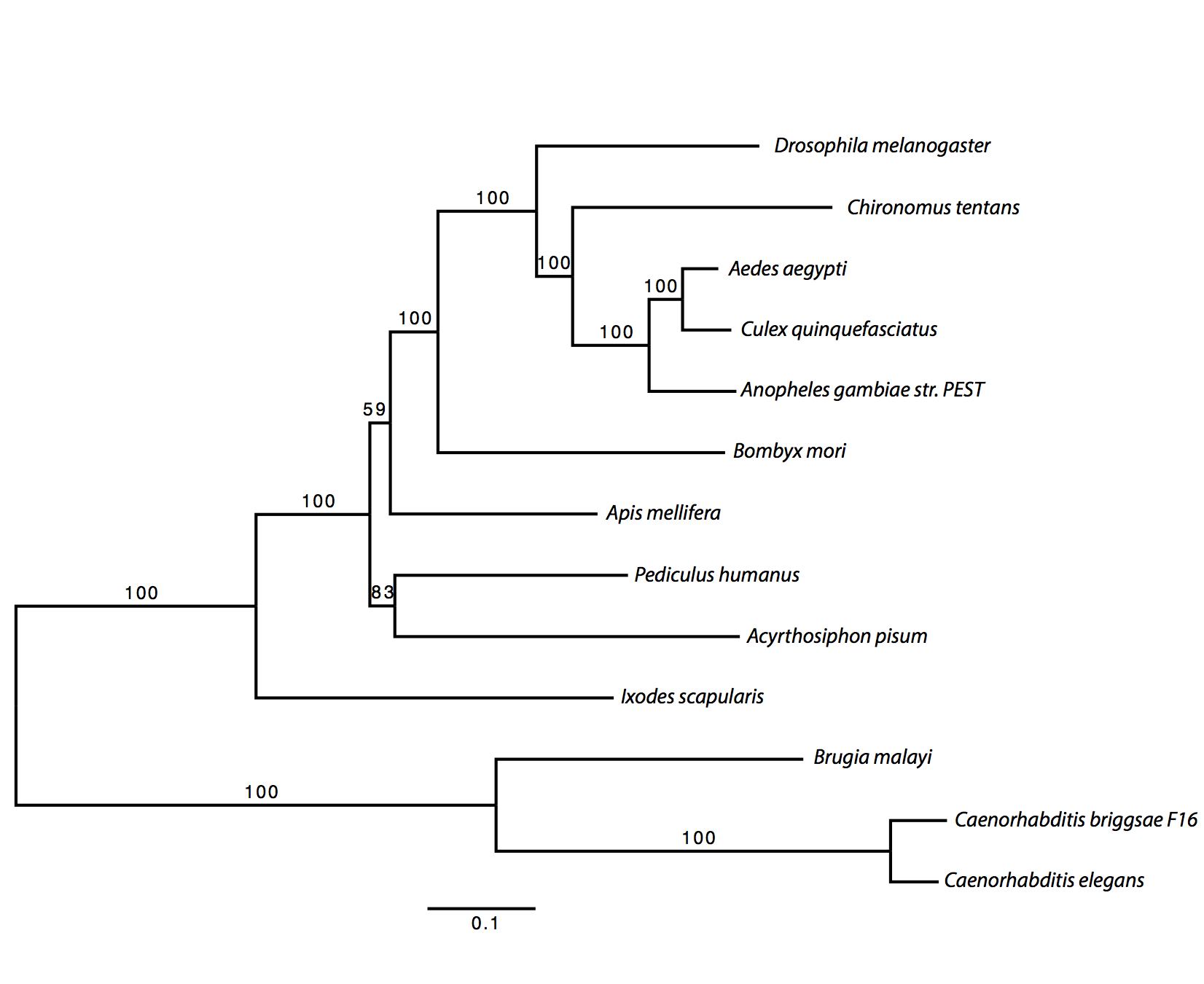
 Figure S2.** A maximum-likelihood phylogenetic tree reconstructed from a concatenation of 531 core gene protein alignments, including 10 arthropods and 3 nematodes. The scale bar depicts amino acid substitutions per site. Branch labels are support values for 100 bootstrap replicates. **Figure S3.** Gene expression values in *Ch. tentans* (log FPKM) versus percent protein identity between potential *D. melanogaster* and *Ch. tentans* orthologs for 16 expression machineries. Each color indicates a set of proteins constituting an expression machinery. FPKM: Fragments Per Kilobase Of Exon Per Million Fragments Mapped.

**
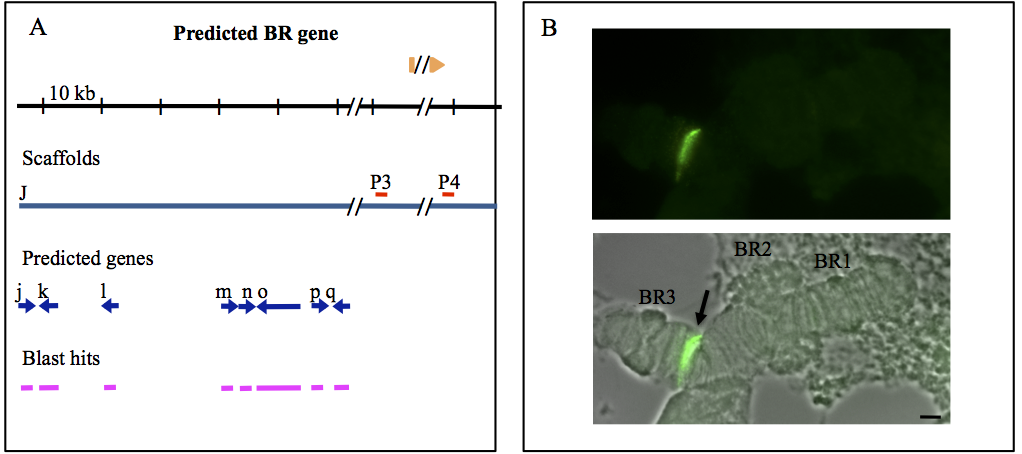
**

**Figure S4.** A) Organization of the predicted BR gene locus. Solid blue line shows assembled scaffold. Two interruptions have been introduced, one in the middle of the predicted gene where sequence information is lacking and one upstream because of space limitations. Dark blue arrows, labelled with small letters, show the locations of predicted genes. Blast hits are shown in pink. P3 and P4 indicate the location of probes used for *in situ* hybridization. B) *In situ* hybridization with probe P3. Probe 4 hybridized to the same chromosomal locus (data not shown). On top, fluorescence image. Below, combined fluorescence and phase contrast image. The hybridization signal was confined to region 5B in chromosome IV. The BR1, BR2 and BR3 loci are indicated for reference.

**SUPPLEMENTARY TABLES**

**Table S1.** Statistics on genome sequencing libraries.

| **Type** | **Insert size** | **Read length** | **Raw  data** | **Filtered data** | **Coverage*** | **Mapped**** |
| --- | --- | --- | --- | --- | --- | --- |
| **DNA-seq** |  |  |  |  |  |  |
| Illumina paired-end | 500 bp | 2 x 100 bp | 5.8 Gbp | 5.6 Gbp | 28X | 90 % |
| Illumina mate-pair | 5 Kbp | 2 x 44 bp | 5.3 Gbp | 4.2 Gbp | 21X | 90 % |
| 454 |  | 400 bp | 0.9 Mbp | 0.8 Gbp | 4X | 68 % |
| Total |  |  | 12.0 Gbp | 10.6 Gbp | 53X | 89 % |
| **RNA-seq** |  |  |  |  |  |  |
| Illumina paired-end | 200 bp | 2 x 101 bp | 11.6 Gbp |  |  | 97 % |

* Filtered data. Based on an estimated genome size of 200 Mbp

** BLAT (92% identity)

**Table S2.** Species distribution of sequences in the NCBI nucleotide database (nt) with homology against a 5% random subset of *Ch. tentans* sequencing reads. Best Blast hits with identity of 98% and >50 bp aligned (454 reads) or with maximum 1 mismatch (Illumina reads) were recorded.

| **Phylum** | Arthropoda | Unclassified | Cnidaria | Nematoda | Mollusca | Others |
| --- | --- | --- | --- | --- | --- | --- |
|  | 93 % | 1 % | 0.7 % | 0.6 % | 0.6 % | 4.1 % |

**Table S3.** Previously identified *Chironomus* repeat sequences added to the *Ch. tentans* *ab initio* repeat library.

| **GI accession number** |
| --- |
| 14531653  156608  156609  156598  3336848  556631  3336845  3002944  2051997  556627  3861491  1448961  14531329  14531331 |

**Table S4. The repeat content of the *Ch. tentans* genome.**

|  | **Repetitive elements** | **Assembly** | **Assembly independent*** |
| --- | --- | --- | --- |
| **Complex repeats** | DNA elements | 0.98 | 1.68 |
|  | SINEs | 0.14 | 0.25 |
|  | LINEs | 0.25 | 0.46 |
|  | LTR elements | 0.09 | 0.10 |
|  | Satellites | 0.20 | 0.26 |
|  | Unclassified | 5.40 | 8.41 |
| **Simple repeats** | Minisatellites | 2.74 | 3.45 |
|  | Low complexity | 0.40 | 0.33 |

* Estimated from a set of 180,000 454 reads longer than 500 bp.

***Table S5. Species included in the OrthoMCL-DB gene family analysis and the phylogenetic reconstruction.***

| **Species** | **Phylum** |
| --- | --- |
| *Chironomus tentans*  *Acyrthosiphon pisum*  *Aedes aegypti*  *Anopheles gambiae* str. PEST  *Apis mellifera*  *Bombyx mori*  *Culex quinquefasciatus*  *Drosophila melanogaster*  *Pediculus humanus*  *Ixodes scapularis*  *Brugia malayi**  *Caenorhabditis briggsae* F16*  *Caenorhabditis elegans** | Arthropoda  Arthropoda  Arthropoda  Arthropoda  Arthropoda  Arthropoda  Arthropoda  Arthropoda  Arthropoda  Arthropoda  Nematoda  Nematoda  Nematoda |

* Outgroup

**Table S6.** Expression machinery genes in *D. melanogaster* with no detected orthologous sequence in *Ch. tentans*. Identifiers according to FlyBase r5.55.

**Machinery Name Gene Protein Gene length (aa)**

Basal transcription factors Taf12L FBgn0031623 FBpp0077111 139

Basal transcription factors Trf2 FBgn0261793 FBpp0303138 1716

Basal transcription factors nht FBgn0041103 FBpp0289298 246

Chromatin remodelling CG12316 FBgn0036483 FBpp0075405 1189

Chromatin remodelling Atac1 FBgn0031876 FBpp0078990 357

Chromatin remodelling D12 FBgn0027490 FBpp0079312 970

Chromatin remodelling Atac2 FBgn0032691 FBpp0080629 775

Chromatin remodelling Sgf11 FBgn0036804 FBpp0112092 197

Nuclear pore Ulp1 FBgn0027603 FBpp0074462 1514

RNA export Nxf3 FBgn0263232 FBpp0305286 560

Splicing factors CG17764 FBgn0029751 FBpp0070724 273

Splicing factors lost FBgn0263594 FBpp0078561 546

Splicing factors CG17098 FBgn0032276 FBpp0079692 653

Splicing factors CG9684 FBgn0037583 FBpp0081342 643

Splicing factors ymp FBgn0261287 FBpp0310517 187

**Table S7.** For each of the U1, U2, U4, U5 and U6 snRNAs, the *Ch. tentans* scaffolds containing significant sequence match to the *D. melanogaster* genes are listed. *D. melanogaster* contains multiple copies of each type of snRNA gene (listed in the right column together with the length of each gene). The length of the matched *Ch. tentans* regions and the percent identity within the matched regions are shown.

| snRNA | *Ch. tentans*, scaffold (region) | Match length  (% identity) | *D. melanogaster*, query (length) |
| --- | --- | --- | --- |
| U1 | sc2105 (1160-1314)  sc1126 (31031-31185) | 1-156 (80)  1-156 (80)  1-156 (80)  1-156 (80)  1-156 (80)  1-156 (80)  1-156 (80)  1-156 (80)  1-156 (80)  1-156 (80) | U1:21D (164 bp)  U1:82Eb (164 bp)  U1:95Ca (164 bp)  U1:95Cb (164 bp)  U1:95Cc (164 bp)  U1:21D  U1:82Eb  U1:95Ca  U1:95Cb  U1:95Cc |
| U2 | sc4193 (1246-1057) | 1-191 (83)  1-191 (83)  1-191 (84)  1-191 (84)  1-191 (83)  1-190 (83) | U2:14B (192 bp)  U2:34ABa (192 bp)  U2:34ABb (192 bp)  U2:34ABc (192 bp)  U2:38ABa (192 bp)  U2:38ABb (191 bp) |
| U4 | sc237 (102346-102432)  sc357 (82409-82327) | 1-87 (77)  5-86 (81)  5-86 (80)  5-86 (79)  5-86 (79) | U4:25F (148 bp)  U4:38AB (142 bp)  U4:39:B (143 bp)  U4:38AB  U4:39B |
| U5 | sc717 (38264-38363)  sc717 (32690-32794)  sc7116 (1377-1475) | 1-99 (86)  17-96 (90)  17-96 (88)  16-86 (91)  17-96 (90)  16-95 (90)  16-94 (90)  19-107 (88)  1-96 (85)  1-96 (84)  16-84 (91)  1-96 (84)  16-103 (88)  16-94 (90)  1-98 (82)  17-96 (87)  17-95 (88)  16-84 (91)  17-96 (90)  16-95 (87)  16-94 (90) | U5:14B (110 bp)  U5:23D (131 bp)  U5:34A (127 bp)  U5:35D (126 bp)  U5:38ABa (127 bp)  U5:38ABb (127 bp)  U5:63BC (123 bp)  U5:14B  U5:23D  U5:34A  U5:35D  U5:38ABa  U5:38ABb  U5:63BC  U5:14B  U5:23D  U5:34A  U5:35D  U5:38ABa  U5:38ABb  U5:63BC |
| U6 | sc7078 (2259-2365)  sc295 (9373-9268)  sc96 (18372-18476) | 1-107 (100)  1-107 (100)  1-107 (100)  1-107 (98)  1-107 (98)  1-107 (98)  3-107 (97)  3-107 (97)  3-107 (97) | U6:96Aa (107 bp)  U6:96Ab (107 bp)  U6:96Ac (107 bp)  U6:96Aa  U6:96Ab  U6:96Ac  U6:96Aa  U6:96Ab  U6:96Ac |

**Table S8.** Oligonucleotides used for in situ hybridization experiments. P1-P4 oligonucleotides were used as PCR primers to obtain probes for in situ hybridization. The BR2.1 and BR2.2 oligonucleotides were labeled with CY3 at their 5′-ends.

| P1a | 5′ GATTGTGGTGTTTAGTACTAGCC 3′ |
| --- | --- |
| P1b | 5′ CATTTGCGAACCATGGTCTGC 3′ |
| P2a | 5′ ACAAATGTCTTATGCAGTCTAG 3′ |
| P2b | 5′ AAACCAACATTTCAGCTTG 3′ |
| P3a | 5′ GTTTGACGTCAATTTGACAG 3′ |
| P3b | 5′ CATAAATGACACGTCGATC 3′ |
| P4a | 5′ ATCGACACCACTATATGAAG 3′ |
| P4b | 5′ TGAGGAAATCATTCATGC 3′ |
|  |  |
| BR2.2 | 5′-CY3-CTCTGGTTTAATTCCTGACCAACTTGGTCT 3′ |
| BR2.1 | 5′-CY3-ACTTGGCTTGCTGTGTTTGCTTGGTTTGCT 3′ |
